# Non-intuitive clustering of 9,10-phenanthrenequinone on Au(111)

**DOI:** 10.3762/bjnano.8.181

**Published:** 2017-08-30

**Authors:** Ryan D Brown, Rebecca C Quardokus, Natalie A Wasio, Jacob P Petersen, Angela M Silski, Steven A Corcelli, S Alex Kandel

**Affiliations:** 1 Department of Chemistry and Biochemistry, University of Notre Dame, Notre Dame, IN 46556 USA; 2 Department of Chemistry, University of Connecticut, Storrs, CT 06269 USA; 3 Department of Chemistry, Tufts University, Medford, MA 02155 USA

**Keywords:** metastable clusters, 9,10-phenanthrenequinone, scanning tunneling microscopy, self-assembly

## Abstract

The direct injection of a 9,10-phenanthrenequinone in tetrahydrofuran solution on a Au(111) substrate in high vacuum results in the formation of metastable clusters with a non-intuitive structure. Metastable, rectangular tetramers of this molecule form in which the net molecular dipoles all orient toward the center of the cluster. This structure does not allow for additional hydrogen bonding and thus the origin of its metastability is not clear. We compare this feature to other structures observed on this surface, as well as those formed during the deposition of 9-fluorenone, which does not exhibit this anomalous clustering behavior.

## Introduction

The goal of crystal engineering is to utilize a combination of intermolecular interactions, molecule–substrate interactions and growth conditions to produce a desired mesoscale or nanoscale structure through self-assembly [1–2]. Generally, this involves a careful selection of these interactions to produce an equilibrium supramolecular assembly that has the desired two- or three-dimensional structure [3–9]. While this technique has been shown to be quite effective at achieving this goal, it is limited to geometries allowed by crystalline structures. Self-assembly under conditions far from equilibrium, under kinetic control, can produce a variety of supramolecular structures not available through equilibrium growth techniques [10–11]. Recently, non-equilibrium growth conditions combined with competing hydrogen bonding elements have been shown to produce supramolecular conformations not formed under thermodynamically controlled growth conditions, including clusters with pentagonal symmetry [12–15] and quasicrystalline assemblies of these pentagonal subunits [16]. In order to exploit non-equilibrium growth methods to produce nanoscale structures, the origin of these metastable species needs to be investigated. An improved understanding of these processes not only allows for the creation of nanoscale structures with non-equilibrium geometries, but also might improve our understanding of polymorphism in organic crystals [17–18].

**Figure 1:**
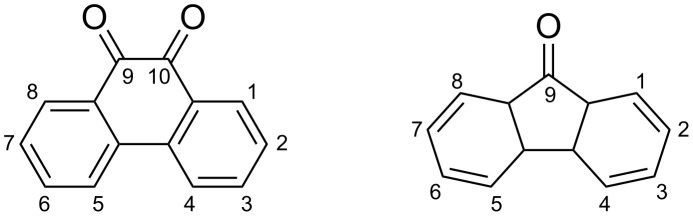
9,10-phenanthrenequinone (left) and 9-fluorenone (right).

Some hydrogen-bonding organic molecules have a common feature in that C–H···O bonding plays a critical role in stabilizing metastable clusters [13–14,19–20]. This makes 9,10-phenanthrenequinone (Figure 1) an attractive target for study, since it has two carbonyl hydrogen-bonding acceptors and multiple aromatic hydrogen donors on the fused ring structure. Possibly as a result, there are six reported polymorphs of the bulk crystal structure [21]. Additionally, the asymmetry of the phenanthrene ring should allow for the determination of the molecular orientation during scanning tunneling microscopy experiments. This molecule has been studied in the past for its role in surface passivation of semiconductor interfaces [22] and its assembly behavior at the liquid–solid interface on graphite [23] but not as extensively on metal surfaces.

Scanning tunneling microscopy is well suited for interrogating large supramolecular structures, as well as determining the structure and orientation of individual molecules at a solid interface [24–26]. This study utilizes scanning tunneling microscopy to demonstrate that the pulse deposition of 9,10-phenanthrenequinone produces metastable clusters, which do not form due to hydrogen bonding considerations alone. They rather have a non-intuitive internal structure, which likely arises after adsorption of a cluster formed in solution. This behavior is not observed after the pulse deposition of 9-fluorenone (Figure 1), which indicates that the origin of this feature must be related to the molecular structure of 9,10-phenanthrenequinone.

## Results and Discussion

**Figure 2:**
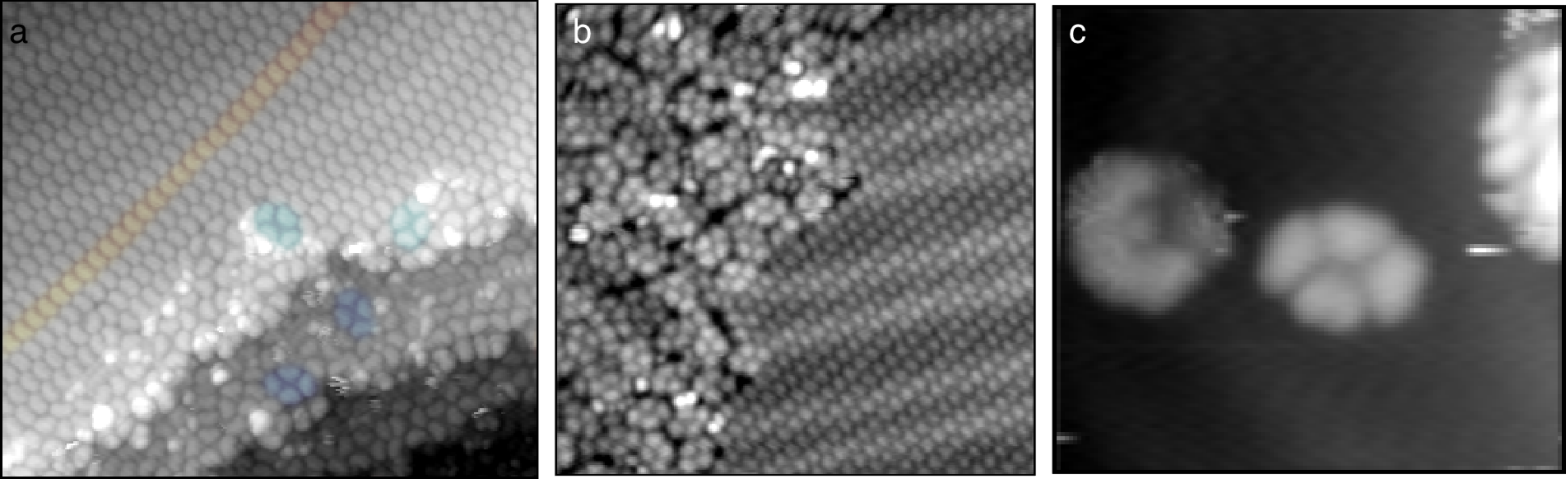
a) STM topography image, 250 Å × 250 Å, of 9,10-phenanthrenequinone on Au(111), with a representative row (red) and some representative tetramers (blue) highlighted. b) STM topography image, 250 Å × 245 Å, of a boundary of tetramers and ordered rows on a single terrace. c) A 79 Å × 72 Å topography image (20 pA, +1.00 V) of an isolated tetramer in a low-coverage region of the surface.

Pulse deposition usually results in a heterogeneous surface, most likely due to the fact that any cluster formation that occurs in the rapidly evaporating droplet will proceed under non-equilibrium conditions and thus can produce kinetic intermediates [13–14,19,27–30]. For 9,10-phenanthrenequinone we observe three broad categories of assembly: disordered molecules, rectangular tetramers, and ordered linear rows, as seen in Figure 2. The crescent- (or banana-)shaped appearance of molecular features suggests that molecules adsorb with the conjugated π-system parallel to the surface.

In the bulk crystal structures of 9,10-phenanthrenequinone, the carbonyls of one molecule form C–H···O bonds with the hydrogens on the rear of the fused ring of another molecule, resulting in row-like bonding motifs. Of the two ordered structures observed on the surface, the linear rows are the feature that most strongly resemble the reported bulk crystalline structures. For some polymorphs, these rows line up exactly such that the molecule is oriented in the direction of the row propagation, while the other reported polymorphs have the molecular orientation offset such that there is a tilt of the molecule relative to the direction of the row [21]. The resulting difference in spacing of neighboring molecules (approximately 0.3 Å) is below the accuracy of our imaging, but the orientation of molecules relative to the direction of the row is easily observable. Our calculations indicate that the pairwise binding energy of these two motifs are essentially isoenergetic (−15.75 kJ/mol for the tilted motif and −15.55 kJ/mol for the tilted and end-to-end orientations, respectively), which could be the reason why both are present in the reported crystal structures.

**Figure 3:**
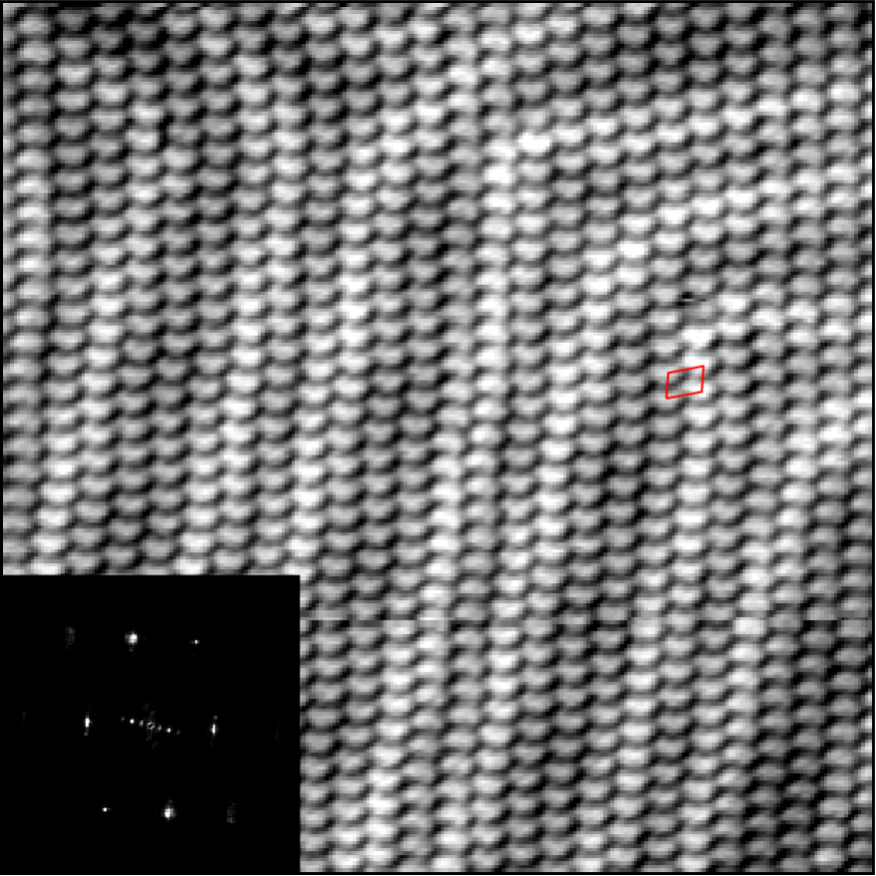
A 237 Å × 237 Å STM topography image acquired at a tip–sample bias of −0.5 V, and a 5 pA setpoint. This image is a large array of 9,10-phenanthrenequinone rows, with the Au(111) herringbone visible underneath. The inset is the 2D Fourier transform of the image, and the red overlay shows the periodicity obtained from the 2D FFT.

Figure 3 shows an area of the surface completely covered by rows of 9,10-phenanthrenequinone. Positional order is evident in both the regular appearance of the lattice and the sharpness of the Fourier transform, and the pseudo-unit cell obtained from this FT gives a rhombohedral unit cell with axes of 7.0 Å along a row, and 9.8 Å between rows, with an angle of 75° between the two axes. This spacing is approximately 1 Å shorter than predicted from bulk crystal structures, but this is likely due to uncertainty in the lateral calibration and possible drift or warping in the image.

A careful inspection of the orientation of molecules within the rows in Figure 3 reveals that neighboring rows can have two orientations. There appears to be a preference for adjacent rows to align in the same direction, but there are also several flips across the area imaged, with no apparent periodicity for this reversal of orientation. The monolayer structure is therefore not actually crystalline, but rather a domain of very stable rows that are positionally ordered. The bulk crystal structures do have alternating directions in adjacent rows, and for a given row-bonding motif the polymorphs are simply different packing of these rows. However, there is no obvious facet of any of these structures that would result in this apparently random pattern, although faceting of a high-index crystal plane has not been ruled out at this time.

**Figure 4:**
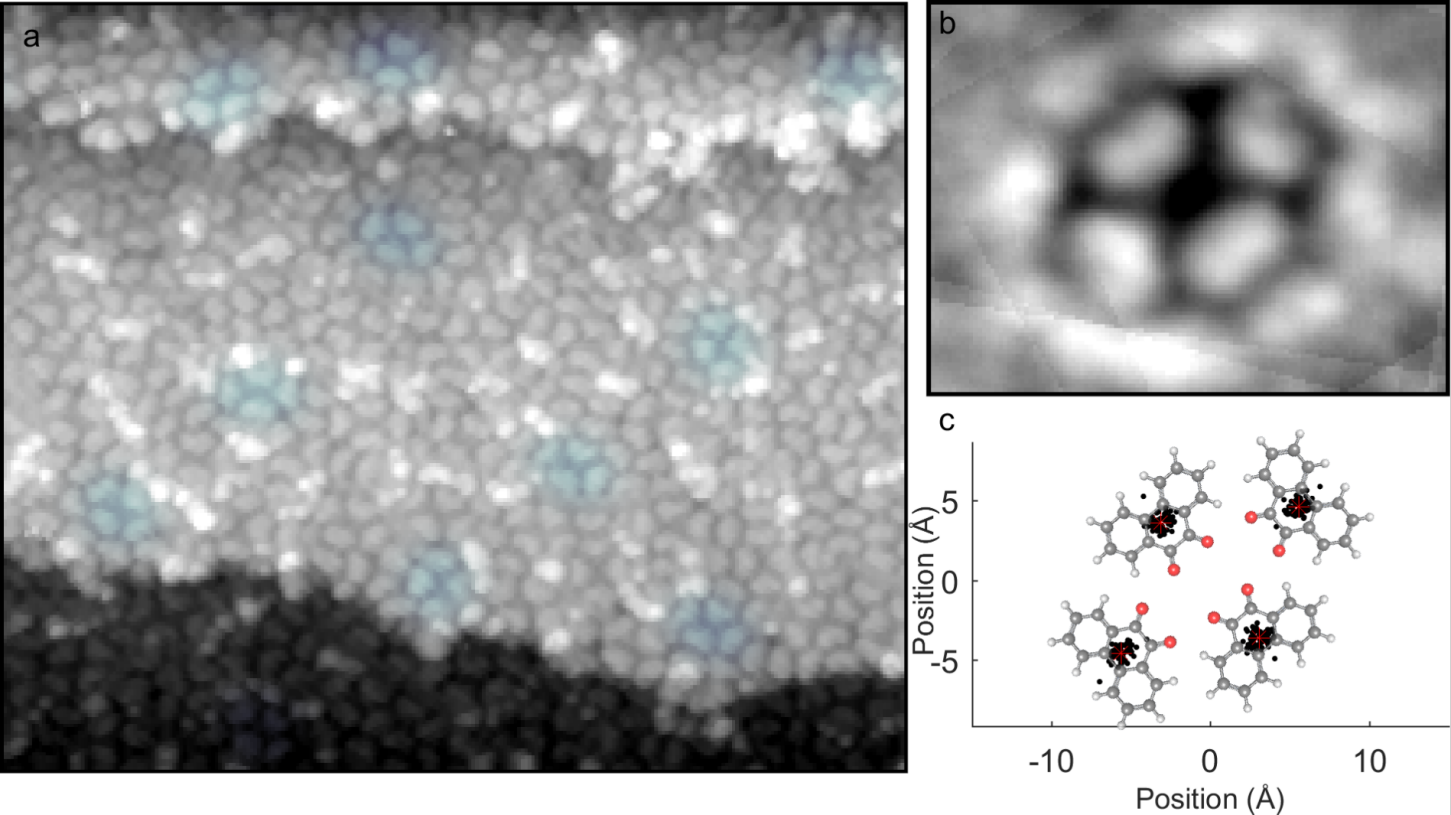
a) A 250 Å × 212 Å STM topography image containing a region of disordered molecules and rectangular tetramers, with some representative tetramers highlighted in blue. A composite image of the 59 fully-resolved tetramers present in this image is shown in panel b), with the proposed molecular conformation as an overlay. The average position of each molecular centroid (red asterisk) is plotted with the actual measured position of each centroid (black) for these 59 clusters in c).

The tetramer, in contrast, does not appear to have any intermolecular bonding motif observed in the crystal. Figure 4 shows an area of high tetramer density, along with some disordered species. Adjacent molecules have centroids approximately 8.6 to 8.7 Å apart and are oriented 90° apart from each other. Opposite molecules are 14.5 Å and 9.5 Å apart from each other for the long and short diagonals, respectively, and are oriented 180° with respect to each other. It is apparent from the original image and the tetramer composite image that within this cluster the molecules orient with the carbonyl groups projecting into the center of this cluster.

If considered as a gas-phase species, the observed tetramer conformation positions the net dipole of each molecule so that it is oriented toward the center. This is not an energetically favorable configuration from an electrostatic standpoint. While in some cases weak dipoles on coinage metals can direct self-assembly [31], a combination of charge transfer and screening of in-plane adsorbate dipoles can result in dipole–dipole interactions being overwhelmed by stronger intermolecular interactions (i.e., van der Waals forces and hydrogen bonding) during growth processes [32–35].

However, the arrangement of molecules in the tetramer does not allow for half of the carbonyl groups present to form C–H···O bonds. Hence, it is difficult to see how this would be a favorable conformation for a cluster formed on the surface. The tetramers are found both on terraces and step edges, and in regions of high and low coverage, as seen in Figure 2; while it is possible that step edges and defect sites play some role in nucleating aggregations of these structures, the role of defects does not seem to be a controlling one. Instead, we propose the explanation that tetramers initially form in solution, either in the rapidly evaporating droplet in transit or a rapidly evaporating film at the Au(111) surface. Given past studies that have observed both direct [30] and indirect [13–14,19] evidence of the formation of metastable clusters in solution during pulse deposition, we think that this mechanism is a plausible one for the formation of these tetramers.

In solution, clusters of four molecules could adopt a metastable 3D configuration with more favorable dipole–dipole and hydrogen-bonding interactions. Then, precipitation onto the Au(111) surface could force it into the observed two-dimensional configuration, where it could be kinetically trapped. If this mechanism is indeed responsible for the presence of these tetramers, then it might be an exploitable method for producing nanoscale structures via kinetically controlled self-assembly.

**Figure 5:**
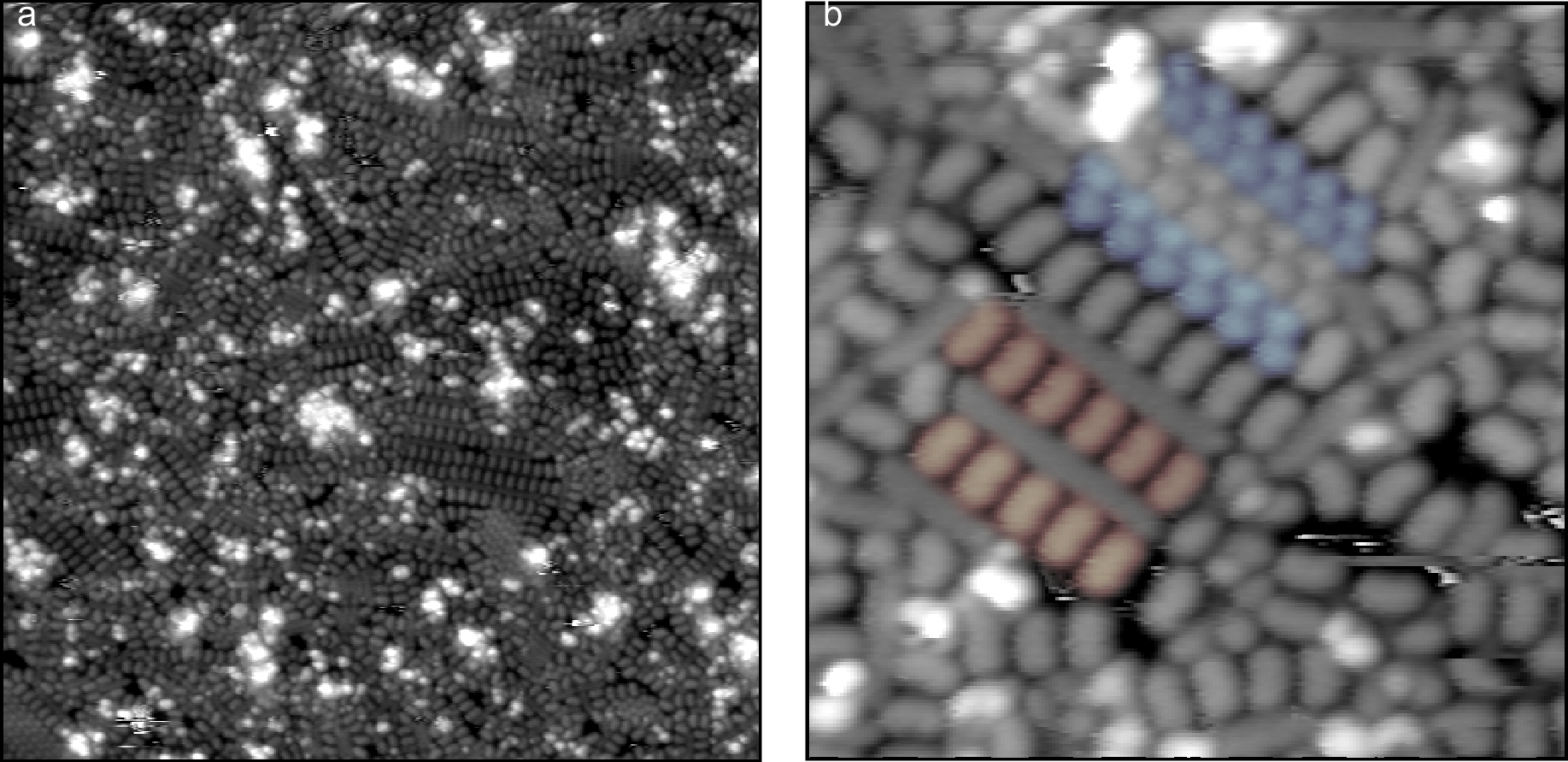
a) A 500 Å × 500 Å STM topography image of 9-fluorenone on Au(111). b) A 110 Å × 109.5 Å STM topography image containing examples of the linear row structures (red), and regions of a close-packed domain (blue).

These experiments were repeated using 9-fluorenone in order to test the role of molecular geometry in determining the metastable species produced during pulse deposition. This molecule did not exhibit any tetramer clusters, and this absence suggests that those anomalous features might be particular to 9,10-phenanthrenequinone. The three main molecular features of this surface are disordered molecules, linear rows, and close-packed domains, as seen in Figure 5.

When comparing the two ordered features of this surface, it is useful to consider the only reported crystal structure of 9-fluorenone. The bulk crystal structure is comprised of 9-fluorenone dimers, slightly offset and oriented 180° apart so that the carbonyls face the hydrogen atoms at the 1- or 8-positions of the aromatic rings for each molecule [36]. This is very different from the binding motifs present in the polymorphs of 9,10-phenanthrenequinone, and the fact that this molecule can form dimers with this orientation might be the reason that tetramers do not form. There might be little to no kinetic barrier to dimer formation for two molecules adopting the orientation needed to form a tetramer. Within the linear rows, the molecules have a spatial periodicity of approximately 7.4 Å apart, and are oriented in the same direction of the row propagation. This implies that the C–H···O bonding occurs between the carbonyl and hydrogen atoms of the 4 and 5 positions for each molecule in the row, save the end molecules. This model is in agreement with the density functional theory calculations, which predict a 7.7 Å periodicity for this binding motif, and find it to be more stable than hydrogen-bonding motifs involving the hydrogens at the 3,4- and 2,3-positions by at least 2.3 kJ/mol.

The identity of the close-packed regions (blue in Figure 5a) is less clear. Assuming that each bright circular region represents one of the benzene rings of the fused heterocycle, the width of these molecules are consistent with the 9-fluorenone molecules in the linear rows and disordered regions, and the close-packed regions have a similar orientation to those in the crystal structure. However, the spatial periodicity between these bi-lobed features is approximately 6.6 Å, which is roughly 90% of the spacing between the molecules of the linear dimer row. The spacing in the bulk dimer is 8.5 Å, and thus this feature is packed too closely to represent a planar dimer structure with the binding motif of the bulk crystal. Even accounting for inaccuracy in the STM imaging, the close-packed periodicity should be larger than the linear rows by about 0.5 Å if this feature actually was comprised of 9-fluorenone dimers. Another bonding motif of 9-fluorenone is a catemer, in which each molecule binds to two other molecules in the structure, in a co-crystal with perfluoro-*ortho*-phenylmercury [37]. The orientation of the molecules in this catemer does not match those in the close-packed domains on this surface, and thus this is also not a plausible assignment for this feature. Another possibility is that this feature consists of molecules that do not adsorb with the π-rings parallel with the surface plane, i.e., there is some tilt associated with their adsorption geometry, but the imaging data is insufficient to strongly support this assignment, or any conclusive assignment, at this time.

**Figure 6:**
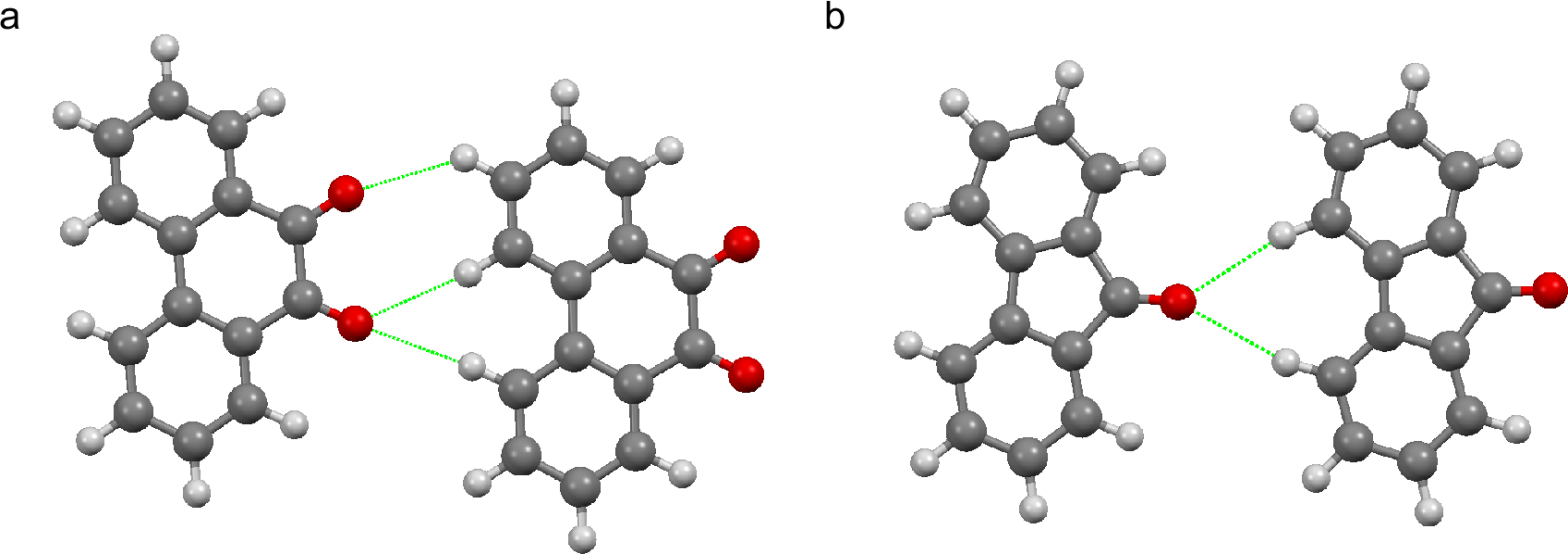
The calculated structure of pairwise interactions in linear rows for (a) 9,10-phenanthrenequinone and (b) 9-fluorenone with the C-H···O hydrogen-bond contacts indicated in green.

The linear rows have less positional order than the 9,10-phenanthrenequinone rows, quite possibly due to the weaker total intermolecular interactions directing the assembly of the rows. It is possible to observe kinks within the rows, which were not present to a noticeable degree in the 9,10-phenanthrenequinone rows. Density functional theory calculations find that the pairwise binding energies are −15.75 kJ/mol and −7.53 kJ/mol for the observed 9,10-phenanthrenequinone rows and 9-fluorenone rows, respectively. Figure 6 displays the calculated structure of pairwise interactions in the linear rows for both molecules. It is evident that the 9,10-phenanthrenequinone dimer has more hydrogen-bonding contacts and is, thus, more stable than that of 9-fluorenone. This disparity might be the reason for a higher fidelity of orientation in linear 9,10-phenanthrenequinone rows relative to 9-fluorenone.

## Conclusion

Pulse deposition of 9,10-phenanthrenequinone on Au(111) results in two types of ordered structures: Ordered linear rows of molecules the bonding motif of which strongly resembles that of some bulk crystal polymorphs, and rectangular tetramers, which have a non-intuitive intermolecular conformation. These tetramers do not appear to be stabilized by competing hydrogen-bonding elements, but do exist as metastable species at this interface. A comparison with the assembly behavior of 9-fluorenone further complicates this picture, in that no tetramers were observed after pulse deposition. 9-fluorenone has two types of ordered feature after pulse deposition: linear rows and close-packed arrays. The linear rows all contain molecules oriented end-to-end in the direction of the row propagation, while the close-packed arrays resemble the orientation of dimers observed in the crystal structure, but have a periodicity inconsistent with any known hydrogen-bonding motif for this molecule. This study demonstrates the importance of developing an improved understanding of self-assembly proceeding under kinetically controlled growth conditions. The ultimate goal of studying non-equilibrium self-assembly is to use both intermolecular interactions and deposition technique to “engineer” metastable states of a given supramolecular conformation. Non-intuitive results such as the 9,10-phenanthrenequinone tetramer suggest that these considerations need to account for the environment in which metastable species evolve.

## Experimental

The Au(111)-on-mica substrates were heated to approximately 350 to 400 °C, then cleaned via three 15 min cycles of argon sputtering followed by a 15 min annealing. The substrates were allowed to cool to room temperature prior to deposition. The samples were produced by preparing a 2 mg/mL solution of either 9,10-phenanthrenequinone or 9-fluorenone in tetrahydrofuran and injecting microliter droplets of this solution at a clean Au(111)-on-mica substrate in a high-vacuum preparation chamber using a solenoid pulse valve (Parker Instruments 9-series, 0.5 mm nozzle diameter, with an IOTA ONE controller). These conditions were sufficient for producing a high-quality substrate and near-monolayer coverages of molecules after deposition.

The samples were then transferred to a cryogenically cooled STM (Omicron LT-STM) in an ultrahigh-vacuum chamber, and imaged once the temperature had equilibrated at 77 K. Typical imaging conditions used were a 10 pA tunneling setpoint with a tip–sample bias of +1.00 V, unless otherwise noted, and used a mechanically-cut Pt/Ir tip.

All calculations were performed using the Q-Chem software package. Structures were optimized with density functional theory (DFT) utilizing the B3LYP functional [38–39]. The 6-311++G(3df,3pd) basis set was employed with a Lebedev quadrature containing 100 radial shells with 302 angular points. Dimers of both species were originally generated to replicate experimental results, but multiple orientations were also created to determine the relative energies of binding motifs that deviate from the experimentally observed supramolecular structure. To address basis-set superposition error, the Boys and Bernardi counterpoise correction was applied to all systems.
